# Computer simulations of neural mechanisms explaining upper and lower limb excitatory neural coupling

**DOI:** 10.1186/1743-0003-7-59

**Published:** 2010-12-10

**Authors:** Helen J Huang, Daniel P Ferris

**Affiliations:** 1 Department of Biomedical Engineering, Human Neuromechanics Laboratory, University of Michigan, 401 Washtenaw Ave., Ann Arbor, MI, 48109-2214, USA; 2 School of Kinesiology, University of Michigan, Ann Arbor, MI, USA; 3 Department of Physical Medicine and Rehabilitation, University of Michigan, Ann Arbor, MI, USA

## Abstract

**Background:**

When humans perform rhythmic upper and lower limb locomotor-like movements, there is an excitatory effect of upper limb exertion on lower limb muscle recruitment. To investigate potential neural mechanisms for this behavioral observation, we developed computer simulations modeling interlimb neural pathways among central pattern generators. We hypothesized that enhancement of muscle recruitment from interlimb spinal mechanisms was not sufficient to explain muscle enhancement levels observed in experimental data.

**Methods:**

We used Matsuoka oscillators for the central pattern generators (CPG) and determined parameters that enhanced amplitudes of rhythmic steady state bursts. Potential mechanisms for output enhancement were excitatory and inhibitory sensory feedback gains, excitatory and inhibitory interlimb coupling gains, and coupling geometry. We first simulated the simplest case, a single CPG, and then expanded the model to have two CPGs and lastly four CPGs. In the two and four CPG models, the lower limb CPGs did not receive supraspinal input such that the only mechanisms available for enhancing output were interlimb coupling gains and sensory feedback gains.

**Results:**

In a two-CPG model with inhibitory sensory feedback gains, only excitatory gains of ipsilateral flexor-extensor/extensor-flexor coupling produced reciprocal upper-lower limb bursts and enhanced output up to 26%. In a two-CPG model with excitatory sensory feedback gains, excitatory gains of contralateral flexor-flexor/extensor-extensor coupling produced reciprocal upper-lower limb bursts and enhanced output up to 100%. However, within a given excitatory sensory feedback gain, enhancement due to excitatory interlimb gains could only reach levels up to 20%. Interconnecting four CPGs to have ipsilateral flexor-extensor/extensor-flexor coupling, contralateral flexor-flexor/extensor-extensor coupling, and bilateral flexor-extensor/extensor-flexor coupling could enhance motor output up to 32%. Enhancement observed in experimental data exceeded 32%. Enhancement within this symmetrical four-CPG neural architecture was more sensitive to relatively small interlimb coupling gains. Excitatory sensory feedback gains could produce greater output amplitudes, but larger gains were required for entrainment compared to inhibitory sensory feedback gains.

**Conclusions:**

Based on these simulations, symmetrical interlimb coupling can account for much, but not all of the excitatory neural coupling between upper and lower limbs during rhythmic locomotor-like movements.

## Background

Central pattern generators (CPGs) are spinal neural networks that produce rhythmic motor commands. For vertebrate locomotion, they are theorized to consist of two half-centers with reciprocal inhibition [1]. When one half-center is active, the other half is inhibited, producing alternating rhythmic bursts. Key features of central pattern generators are that they can produce rhythmic outputs without rhythmic inputs and they can entrain their rhythmic outputs to sensory feedback. Experimental data on both animals and in humans support the idea that central pattern generators exist. A spinalized cat can be taught to walk after repeated step training [2,3]. In humans, individuals with incomplete and even clinically complete spinal cord injuries can produce rhythmic lower limb motor patterns with appropriate sensory feedback [4-8].

Central pattern generators can be modeled with nonlinear mathematical equations that produce an oscillatory output. The Matsuoka oscillator is one type of mathematical oscillator that has been used to simulate biological oscillators [9-17]. The Matsuoka oscillator consists of two reciprocally inhibited simulated neurons [9,10], similar to the half-center theory of biological central pattern generators [1]. Each neuron receives a tonic input, which corresponds to the tonic descending signal from the midbrain that drives rhythmic output in biological locomotor neural networks [18,19]. Matsuoka oscillators have been applied to simulate neuromechanical control of bio-inspired robots [13-15] and computer models of biomechanical bodies [16,17,20]. Previous modeling studies inter-connecting neural oscillators have investigated coupling effects on frequency, phasing, synchronization, and coordination of oscillator outputs [21]. However, we are unaware of any models of inter-connected neural oscillators that focus on changes in oscillator amplitude.

We are interested in understanding the role of inter-oscillator connections on oscillator output because it may provide greater insight about interlimb neural coupling observed in humans. Experiments on humans have shown that upper limb movement and muscle recruitment can alter lower limb muscle activation [22,23]. Specifically, greater upper limb effort increases muscle activation of passive lower limbs in neurologically intact individuals [24-26] and individuals with incomplete spinal cord injuries [27] during a rhythmic upper and lower limb movement task. Conversely, active lower limb effort also increases passive upper limb muscle activation [25,27]. Upper limb movement can also alter lower limb muscle activation patterns in individuals with incomplete spinal cord injuries during a standing reciprocal leg swing task [28] and in individuals with stroke during treadmill training [29]. Additionally, clinical observations suggest that reciprocal arm swing increases and improves muscle activation in individuals with spinal cord injuries [8,30]. The neural mechanisms responsible for these interlimb excitatory effects are difficult to determine in humans.

One approach for investigating the neural mechanisms involved in the experimental observations is to model the neural pathways. The purpose of this computer simulation study was to test potential neural mechanisms that may explain excitatory interlimb coupling in humans. We hypothesized that interlimb spinal pathways could not account for the levels of muscle recruitment enhancement revealed in our previous experimental studies [25]. Believing in the principle that the simplest model that can explain an observed behavior provides key insight into the dynamics [31], we aimed to create the simplest model possible that still faithfully reproduced the most important behavioral observations from our previous studies. We used a Matsuoka oscillator to model the central pattern generator for each limb. To understand the effects of interlimb coupling on output enhancement, we used a systematic approach, beginning with a single CPG model, then a two-CPG model, and lastly a four-CPG model. We first determined behavioral principles associated with increasing sensory feedback gains and frequencies for enhancing CPG output in a single CPG. We then tested a two-CPG model to determine the effect of coupling flexors to flexors and extensors to extensors (flexor-flexor/extensor-extensor) versus crossing the connections to couple flexors with extensors (flexor-extensor/extensor-flexor). Lastly, we interconnected four Matsuoka oscillators to test the effects of different combinations of inhibitory and/or excitatory interlimb pathways.

## Methods

### Matsuoka oscillators

**Figure 1:**
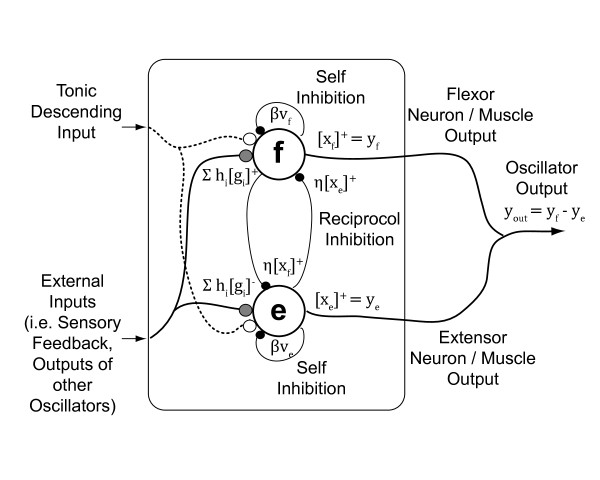
**Schematic of a Matsuoka oscillator**. Two neurons, a flexor (f) and an extensor (e), reciprocally inhibit each other. External inputs (*g*_i_) such as sensory feedback or inputs for other neurons can be either inhibitory or excitatory, depending on the gain (*h*_i_). Black circles indicate inhibitory inputs. White circles indicate excitatory inputs. Gray circles can be either inhibitory or excitatory.

We modeled each limb's central pattern generator using a Matsuoka oscillator (Figure 1) with the following governing equations:

(1) $$\tau_{1}{\dot{x}}_{i,f}=c_{i}-x_{i,f}-\beta v_{i,f}-\eta {\left[x_{i,e}\right]}^{+}-{\sum_{j=1}^{n}h_{j}\left[g_{j}\right]}^{+}$$

(2) $$\tau_{2}{\dot{v}}_{i,f}=-v_{i,f}+{\left[x_{i,f}\right]}^{+}$$

(3) $$\tau_{1}{\dot{x}}_{i,e}=c_{i}-x_{i,e}-\beta v_{i,e}-\eta {\left[x_{i,f}\right]}^{+}-{\sum_{j=1}^{n}h_{j}\left[g_{j}\right]}^{-}$$

(4) $$\tau_{2}{\dot{v}}_{i,e}=-v_{i,e}+{\left[x_{i,e}\right]}^{+}$$

(5) $$y_{i,f}={\left[x_{i,f}\right]}^{+}$$

(6) $$y_{i,e}={\left[x_{i,e}\right]}^{+}$$

Each flexor (f) and extensor (e) neuron has a firing rate, *x*_i _and an adaptation state, υ_i _where *i *= RU (Right Upper Limb), LU (Left Upper Limb), RL (Right Lower Limb), and LL (Left Lower Limb). The output of the flexor or extensor neuron is *y*_i,f _or *y*_i,e _and is equal to [*x*_i_]^+ ^the positive part of the flexor or extensor neuron firing rate *x*_i_, respectively. Similarly, [*g*_j_]^+ ^is the positive part of the external input and [*g*_j_]^- ^is the negative part of the external input. Each external input has an associated gain, *h*_j_. Possible external inputs include joint angle, limb angle, neuron activity, among others. In the Matsuoka oscillator equations, positive gains provide inhibitory feedback while negative gains provide excitatory feedback. We focused on inhibitory sensory feedback which appears to more faithfully reproduce biological systems. The constant *c*_i _is the tonic descending signal, which represents descending neural drive from the midbrain [18,19]. The *β *constant modulates the strength of self-inhibition and the *η *constant modulates the strength of reciprocal inhibition between the flexor and extensor neurons. *τ*_1 _and *τ*_2 _are time constants that affect the shape and intrinsic frequency of the oscillator.

The baseline parameter values our model were *c *= 2, *β *= 2.5, *η *= 2.5, *τ*_1 _= 0.35, and *τ*_2 _= 0.7, which we set according to previously developed guidelines [14]. Tonic descending input, *c *= 2 produces an oscillator output amplitude of ~1, which made it easier to compare output amplitudes. We set *τ*_1 _and *τ*_2 _to provide an endogenous oscillator frequency of 0.32 Hz, ω_cpg_, which is slower than normal walking step frequencies. For the sensory feedback signal which was analogous to joint angle, we used a sine wave with a frequency of 0.625 Hz, ω_s_, and amplitude of 1. This frequency matched the stepping frequencies we used in our recumbent stepping experimental studies [24,25].

### One-CPG model

Using a single Matsuoka oscillator, we determined the effects of increasing sensory feedback strength and frequency on enhancing oscillator output for a given tonic descending signal, *c *= 2. We set the sensory feedback gain to be *h*_s _= k_s_*c, which was relative to the tonic descending drive input. Similarly, we set the sensory feedback frequency to be ω_s _= k_ωs_*ω_cpg, _which was relative to the endogenous frequency of the oscillator. Oscillator amplitude enhancement occurred if parameters led to greater oscillator output amplitudes compared to the baseline condition of no sensory feedback, *h*_s _= 0 or ω_s _= 0.

### Two-CPG models

In a two-CPG model, there were two possible coupling geometries: A) connecting the flexor neurons to each other and the extensor neurons to each other (f-f/e-e) and B) connecting the flexor neuron to the extensor neuron of the other oscillator (f-e/e-f). These models represented interlimb coupling pathways between an upper limb CPG and a lower limb CPG. To simulate ipsilateral coupling, *h*_ip_, we set the lower limb CPG sensory feedback, *h*_s lo _= sin(2πω_s_t + π) to be anti-phase with the upper limb CPG sensory feedback, *h*_s up _= sin(2πω_s_t), simulating the anti-phase movement of ipsilateral limbs during locomotion. To simulate contralateral coupling, *h*_c, _we set the sensory feedback of the lower limb CPG to be in-phase with the upper limb CPG, simulating phasing of contralateral upper-lower limb pair during locomotion. The lower limb CPG received no tonic descending drive, *c*_lo _= 0 while the upper limb CPG tonic descending drive was set to *c*_up _= 2. This tested whether interlimb coupling, *h*_ip _or *h*_c_, could result in enhancement of the lower limb CPG. We tested excitatory and inhibitory ipsilateral *h*_ip _gains (or contralateral *h*_c _gains), in combination with either excitatory or inhibitory sensory feedback. Thus, the parameters tested were coupling geometry (f-f/e,e and f-e/e-f), coupling gain (*h*_ip _or *h*_c_), and lower limb sensory feedback gain, *h*_s lo_.

### Four-CPG model

Experimental studies suggest that there is interlimb neural coupling [22,32]. If the primary mechanisms of interlimb neural coupling are spinal connections among the locomotor networks, then interconnecting four Matsuoka oscillators would be a simple representative model. One advantage of computer simulations is that we can test different connection configurations or neural architectures. In a previous experimental study, we showed a preference for ipsilateral neural coupling of flexors and extensors during a locomotor-like movement [25] and predicted that this feature would be inherent in a four-CPG model. We selected coupling geometries based on our two-CPG model results and explored a three dimensional parameter space consisting of bilateral (*h*_b_) gains, contralateral (*h*_c_) gains, and ipsilateral (*h*_ip_) gains. We tested both excitatory and inhibitory coupling gains. We also focused on symmetrical coupling structures such that the gain was the same in both directions (ex. from upper to lower and from lower to upper CPGs). The upper limb CPG tonic descending drive was set to *c*_up _= 2 and the lower limb CPG tonic descending drive was set to *c*_lo _= 0. The lower limb sensory feedback was set to be inhibitory, *h*_s_lo _= 1.

### Simulation and Analysis

We built the model in MATLAB software program (Mathworks, Natick, MA) and performed each simulation with a time step of 0.01 seconds for 20 seconds. We considered oscillator output to be analogous to muscle recruitment and calculated the output frequency and peak amplitude for each oscillator output burst. We defined the period of each burst as the time between consecutive rising edges of output activity. From each period, we calculated an output frequency. To determine muscle recruitment amplitudes, we identified peak values of the output bursts. Enhancement occurred when amplitudes exceeded the amplitude of the baseline condition. We rejected parameter sets that did not demonstrate steady state, alternation of flexor and extensor bursts within an oscillator, correct phasing among oscillators, or entrainment to the sensory feedback frequency. We then compared lower limb CPG enhancement predicted from the models to experimental data that showed enhancement of 50+% passive lower limb muscle recruitment with maximal effort in the upper limbs [24,25,27].

## Results

**Figure 2:**
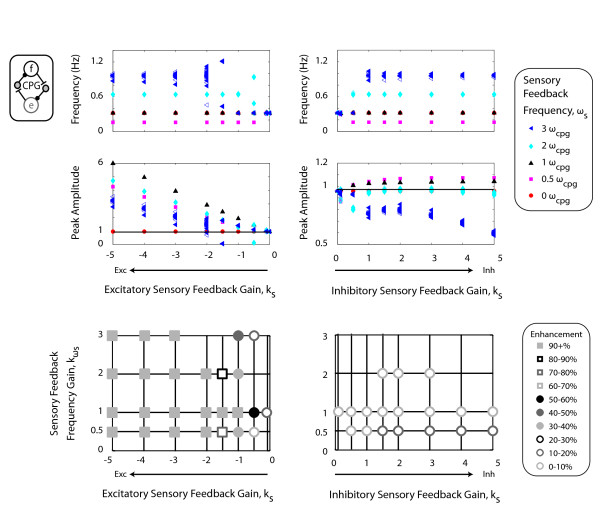
**One-CPG model**. Each symbol represents the frequency and peak amplitude of individual bursts from a single Matsuoka oscillator for different combinations of sensory feedback gains (k_s_) and frequencies (k_ωs_). The equations for the Matsuoka oscillator indicate that negative sensory feedback gains are excitatory and positive sensory feedback gains are inhibitory. Enhancement refers to burst amplitudes greater than the baseline condition of no sensory feedback, *h*_s _= 0. Enhancement amplitudes are shown as percentages of the baseline amplitude of 0.96. Grid intersections indicate parameter combinations tested. Intersections without a symbol indicate that the output behaviour did not reach steady state or did not have alternating flexor and extensor bursts. Sensory feedback gain values tested were 0, 0.1, 0.5, 1, 2, 3, 4, and 5 while sensory feedback frequency gain values tested were 0, 0.5, 1, 2, and 3.

In the one-CPG model, inhibitory sensory feedback gains enhanced oscillator output up to 12% (Figure 2). Enhancement occurred when output amplitude exceeded 0.96, the baseline amplitude of the oscillator with no sensory feedback k_s _= 0 or k_ωs _= 0. For a given inhibitory feedback gain (e.g. k_s _= 1), output amplitudes decreased with increasing sensory feedback frequency. For sensory feedback frequencies less than twice the endogenous frequency, increasing inhibitory sensory feedback gains initially enhanced output and then attenuated output amplitude. For excitatory sensory feedback gains in the one-CPG model, increasing excitatory feedback gains increased amplitude enhancement. For a given excitatory feedback gain (e.g. *h*_s _= -1), maximal enhancement occurred when the sensory feedback frequency matched the endogenous oscillator frequency, k_ωs _= 1 or ω_s _= ω_cpg_.

**Figure 3:**
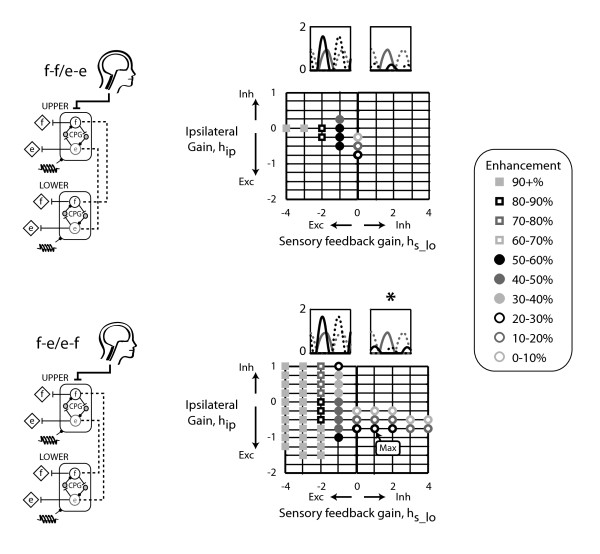
**Ipsilateral two-CPG models**. Two ipsilateral two-CPG models were tested: 1) ipsilateral flexor-flexor/extensor-extensor (f-f/e-e) and 2) ipsilateral flexor-extensor/extensor-flexor (f-e/e-f). Representative time series output bursts for the two-CPG model with either excitatory or inhibitory sensory feedback which produced maximal enhancement. Solid lines are flexor bursts and dotted lines are extensor bursts. * indicate the upper limb bursts (gray line) are out-of-phase with the lower limb bursts (black lines). Enhancement amplitudes are shown as percentages of the baseline amplitude of 0.96. "Max" label indicates maximal enhancement. Grid intersections indicate parameter combinations tested. Intersections without a symbol indicate that the output behaviour did not reach steady state or did not have alternating flexor and extensor bursts. Sensory feedback gain values tested were 0, 1, 2, 3 and 4. Ipsilateral coupling gain values tested were -2 to 1 in increments of 0.25. The helical symbol represents a muscle spindle that signifies sensory feedback.

**Figure 4:**
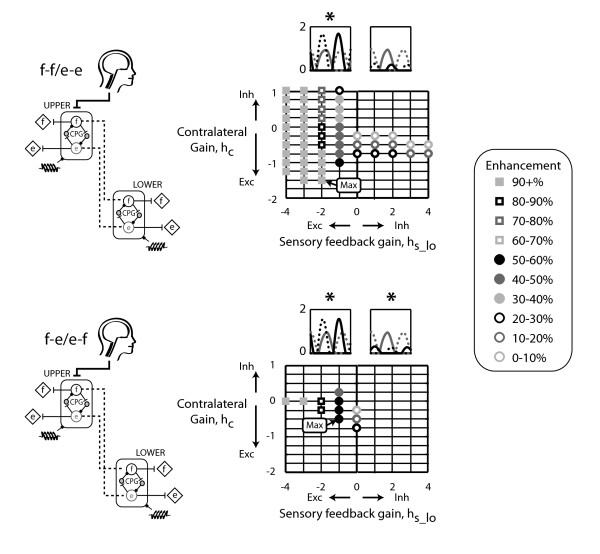
**Contralateral two-CPG models**. Two contralateral two-CPG models were tested: 1) contralateral flexor-flexor/extensor-extensor (f-f/e-e) and 2) contralateral flexor-extensor/extensor-flexor (f-e/e-f). "Max" label indicates maximal enhancement due to excitatory interlimb coupling, where enhancement equalled the maximum amplitude observed within a single excitatory sensory feedback gain minus the amplitude observed with no interlimb coupling gain (i.e. *h*_c _= 0). Other figure details are the same as in Figure 3.

The two-CPG model with inhibitory sensory feedback gains that produced rhythmic bursts in the lower limb CPG that were out-of-phase with the upper limb CPG bursts was the ipsilateral flexor-extensor/extensor-flexor coupling model (Figure 3 *). This model enhanced lower limb CPG amplitude up to 26%. We defined enhancement as the lower limb CPG output divided by the baseline amplitude of 0.96. This baseline amplitude value was the amplitude of the upper limb CPG output and would have been the baseline amplitude of the lower limb CPG if it were to receive the same descending tonic input as the upper limb CPG. The two-CPG models with excitatory sensory feedback gains that produced alternating rhythmic bursting pattern between the upper and lower limb CPGs were the contralateral coupling models (Figure 4 *). The contralateral flexor-flexor/extensor-extensor model generated rhythmic steady state bursting patterns in more of the contralateral gain-sensory feedback gain parameter space than the contralateral flexor-extensor/extensor-flexor model. In the flexor-flexor/extensor-extensor contralateral coupling model, excitatory contralateral gains enhanced lower limb CPG output by up to 20% while in the flexor-extensor/extensor-flexor contralateral coupling model, excitatory contralateral gains enhanced lower limb CPG output by up to 3%. Here, enhancement was defined within a single excitatory sensory feedback gain such that enhancement was due to changes in excitatory contralateral gains, not due to excitatory sensory feedback. Specifically, enhancement within a specific sensory feedback gain equaled the difference between the maximum amplitude observed across excitatory interlimb coupling gains and the baseline amplitude when the interlimb coupling gain was zero. The maximal enhancement due to excitatory interlimb coupling occurred at excitatory sensory feedback *h*_s_lo _= -2 (Figure 4 "max" label). At greater excitatory sensory feedback gains, *h*_s_lo _= -3 and -4, enhancement reached 16% and 13%, respectively.

**Figure 5:**
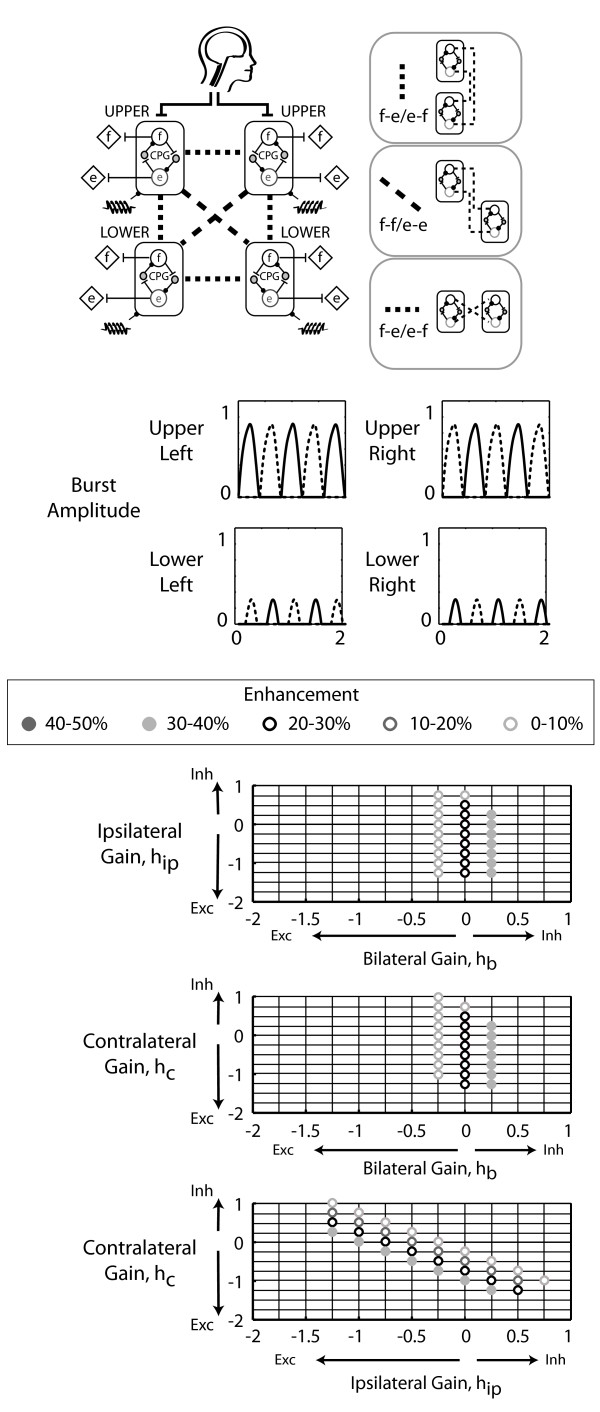
**Four-CPG model with bilateral flexor-extensor/extensor-flexor coupling**. Four CPGs were interconnected to have ipsilateral flexor-extensor/extensor-flexor, contralateral flexor-flexor/extensor-extensor, and bilateral flexor-extensor/extensor-flexor coupling. The helical symbol represents a muscle spindle that signifies sensory feedback. Representative time series output bursts for the four-CPG models indicate that the bursting patterns of contralateral CPGs (upper left and lower right, upper right and lower left) were in-phase while bilateral CPGs (upper left and upper right, lower left and lower right) were out-of-phase. Grid intersections indicate parameter combinations tested. Intersections without a symbol indicate that the output behaviour did not reach steady state or did not have alternating flexor and extensor bursts. Sensory feedback was inhibitory, *h*_s_lo _= 1.

**Figure 6:**
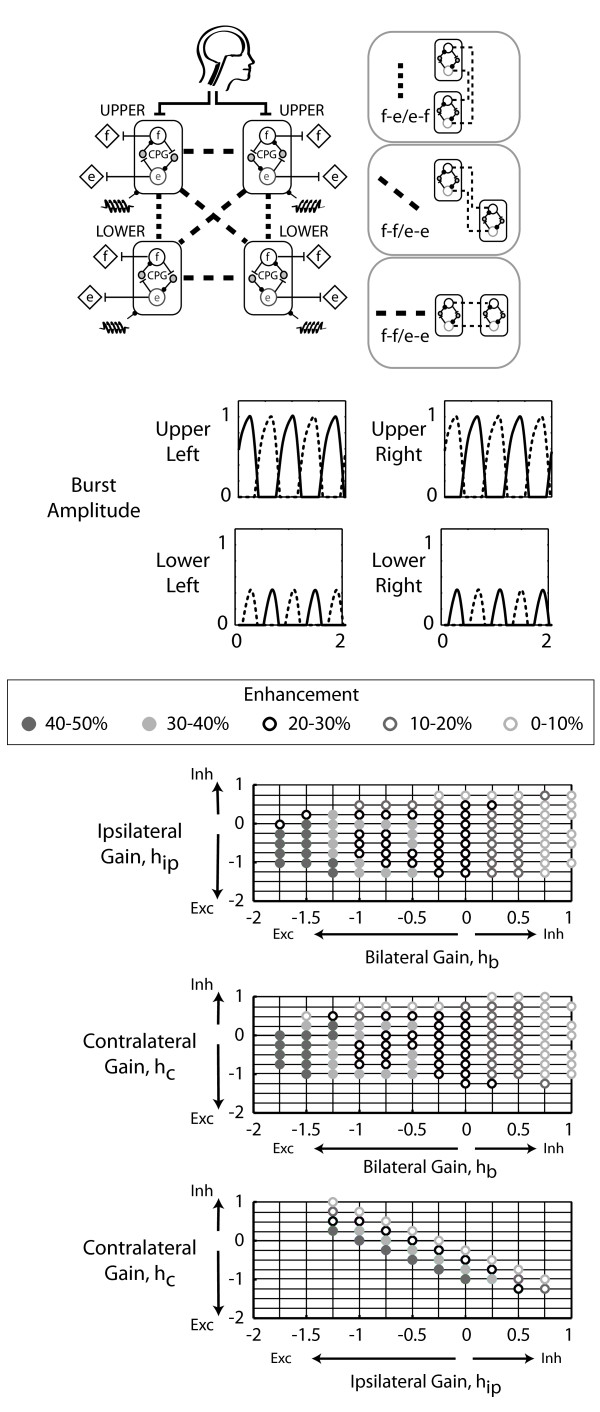
**Four-CPG model with bilateral flexor-flexor/extensor-extensor coupling**. Four CPGs were interconnected to have ipsilateral flexor-extensor/extensor-flexor, contralateral flexor-flexor/extensor-extensor, and bilateral flexor-flexor/extensor-extensor coupling. Figure details are the same as in Figure 5.

Based on the two-CPG models, we interconnected four CPGs to have ipsilateral flexor-extensor/extensor-flexor coupling and contralateral flexor-flexor/extensor-extensor coupling. We then added either bilateral flexor-flexor/extensor-extensor coupling or bilateral flexor-extensor/extensor-flexor coupling. Both models generated alternating flexor and extensor muscle bursts of the upper left and lower right CPGs that were in-phase (Figure 5). Likewise, the upper right and lower left limb flexor and extensor bursts were also in-phase with each other. The muscle recruitment patterns of the upper left and lower right CPG pair were out-of-phase with the burst patterns of the upper right and lower left CPG pair. The ipsilateral flexor-extensor/extensor-flexor, contralateral flexor-flexor/extensor/extensor, and bilateral flexor-extensor/extensor-flexor model enhanced output up to 32% (Figure 5). Maximal enhancement occurred with excitatory ipsilateral and contralateral coupling gains and with inhibitory bilateral coupling. Additionally, this four-CPG model required relatively small interlimb coupling gains (Figure 5). In the ipsilateral flexor-extensor/extensor-flexor, contralateral flexor-flexor/extensor-extensor, and bilateral flexor-flexor/extensor-extensor four-CPG model, enhancement reached levels of up to 46% (Figure 6). However, unlike the bilateral flexor-extensor/extensor-flexor four-CPG model, maximal enhancement occurred with excitatory bilateral coupling gains and at relatively larger bilateral coupling gain values.

## Discussion

These simulations indicated that interlimb coupling can enhance rhythmic steady state muscle recruitment patterns during rhythmic locomotor-like movements. However, the enhancement due to interlimb coupling was limited to < 32%. During a rhythmic locomotor-like task, active reciprocal rhythmic arm exertion can enhance passive lower limb muscle activity by > 32% [24,25,27]. While increasing excitatory ipsilateral, contralateral, and/or bilateral gains in the two-CPG and four-CPG models could provide greater enhancement, gains too large no longer produced rhythmic alternating bursts of the lower limb flexors and extensors. The results from the models and experimental data suggested that excitatory interlimb pathways alone were not sufficient to explain muscle enhancement of unintended muscles.

Interlimb pathways that connect the upper and lower limb locomotor networks likely significantly contribute to excitatory neural coupling. Propriospinal interneurons couple the cervicothoracic to the lumbosacral segments to help coordinate movements of hindlimbs and forelimbs in cats [33,34] and in rats. A study of decerebrate cats walking on a transversely split treadmill revealed that the hindlimbs adapted to changes in forelimb stepping speed; however, the forelimbs did not adapt to changes in hindlimb stepping speed [35]. These results suggested that there were excitatory ipsilateral ascending pathways and inhibitory ipsilateral descending pathways between the flexors of the hindlimb and forelimb locomotor networks [35]. In a neonatal rat spinal cord preparation, pharmacological activation of the hindlimb locomotor neural networks could drive the forelimb locomotor neural networks, but not in the reverse direction [36]. These researchers proposed that caudorostral excitatory pathways help coordinate forelimb and hindlimb movements [36]. We previously demonstrated that in neurologically intact individuals and individuals with incomplete spinal cord injury, excitatory neural coupling was bidirectional [25,27]. Active upper limb effort enhanced passive lower limb muscle recruitment and likewise, active lower limb effort enhanced passive upper limb muscle recruitment. Because of this symmetrical behaviour, we propose that connections between upper and lower limbs act symmetrically. While experimental studies support the existence of interlimb pathways, it is difficult to determine if interlimb pathways are excitatory or inhibitory, symmetrical or asymmetrical, or if they modulate to improve efficacy of the motor patterns for particular movements.

Our models indicate that the simplest case, symmetrical excitatory interlimb coupling, can result in substantial enhancement. All of our simulations had symmetrical coupling gains such that gains from upper to lower limb CPGs were equal to gains from lower to upper limb CPGs. Subsequently, the simulation results had symmetrical bursting patterns. We also simulated a limited set of asymmetrical gains such as excitatory coupling from upper to lower CPGs and inhibitory coupling from lower to upper CPGs. These asymmetrical gains resulted in more asymmetrical and skewed output burst shapes and altered phasing relationships. This suggests that asymmetrical interlimb coupling results in asymmetrical outputs and that symmetrical interlimb coupling results in symmetrical outputs. Inherent with asymmetrical interlimb coupling gains is a need for a gating mechanism to switch from one asymmetrical scheme to another. This added complexity makes asymmetrical interlimb coupling structures seem less likely. Asymmetrical behaviour could arise from other neural mechanisms that act asymmetrically on motor neurons, rather than from asymmetrical interlimb coupling gains. Afferent pathways or supraspinal inputs may act asymmetrically on motor neurons, producing asymmetrical muscle activity patterns.

A few principles emerged from our systematic approach of building upon the results of a single CPG, to two CPGs, and then to four CPGs. The first principle was that ipsilateral coupling acts between flexors and extensors and also prevails with inhibitory sensory feedback (Figure 3 *). The ipsilateral flexor-extensor/extensor-flexor model was the only model to produce anti-phase bursts between the upper and lower limb CPGs when inhibitory sensory feedback gains were used. This preference for ipsilateral flexor-extensor coupling agreed with our previous experimental results. In neurologically intact individuals, upper limb pulling was coupled to ipsilateral vastus medialis and soleus muscle activation, while upper limb pushing activated the ipsilateral tibialis anterior [25]. A second principle was that contralateral coupling probably connects flexors to extensors and prevails with excitatory sensory feedback (Figure 4 *). The models imply that if sensory feedback mechanisms are inhibitory, then excitatory coupling is ipsilateral and if sensory feedback mechanisms are excitatory, then excitatory coupling is contralateral. Our experimental data on neurologically intact individuals demonstrated a preference for ipsilateral coupling which suggests that sensory feedback during our experimental task was inhibitory. Another principle was that the CPGs could entrain to the sensory feedback frequency at smaller inhibitory sensory feedback gains compared to excitatory feedback gains. This provides more support that sensory feedback mechanisms are primarily inhibitory. Interestingly, inhibitory sensory feedback gains could produce small amounts of enhancement, up to 12%. Lastly, enhancement occurred at relatively smaller interlimb coupling gains with the bilateral flexor-extensor/extensor-flexor coupling in the four-CPG than the bilateral flexor-flexor/extensor-extensor coupling model (Figure 5). Thus, we interpreted the bilateral flexor-extensor/extensor-flexor coupling model to be more plausible and hence, conclude that maximal enhancement due to excitatory interlimb coupling in a four-CPG model was 32% (Figure 5).

We analyzed a variety of interlimb coupling gains and neural architectures. Our systematic approach allowed us to justify choices of neural coupling geometry such as using ipsilateral flexor-extensor/extensor-flexor coupling and contralateral flexor-flexor/extensor-extensor coupling in the four-CPG model. We used inhibitory sensory feedback gains, *h*_s_lo _= 1, for the four-CPG models because relatively small inhibitory sensory feedback gains produced in-phase bursts amongst contralateral CPG pairs that were out-of-phase with bilateral and ipsilateral CPG pairs. Surprisingly, excitatory sensory feedback of similar magnitude, *h*_s_lo _= -1, did not produce appropriate phasing among the CPG bursts. Another advantage of using inhibitory sensory feedback gains was that the potential confounding enhancement due to sensory feedback was smaller. We used the same tonic descending input value to the upper limb CPGs of *c*_up _= 2 for all models, which made comparisons easier amongst models. If we increased *c*_up_, both upper and lower limb CPG output amplitudes increased by the same percent. For each model, we tested a parameter space sufficiently large such that the parameter space surrounding maximal enhancement corresponded with either less enhancement or no steady state behaviour. Regardless of the exact amount of maximal enhancement, the models suggested that enhancement due to excitatory interlimb coupling was limited. One possible explanation for the limited effect of excitatory interlimb coupling is that enhancement occurs when specific temporal characteristics coincide and that these occurrences are limited in these non-linear oscillators. We also considered creating more complex models, such as using the Hodgkin-Huxley model for the motor neurons. However, we chose to keep the models as simple as possible to identify inherent interlimb coupling characteristics that could explain the behavioural results.

Based on our simulations that focused on the effects of interlimb coupling, we propose that in addition to excitatory interlimb pathways, supraspinal pathways and/or excitatory afferent feedback can sufficiently explain the levels of enhancement observed in our experimental results. One potential supraspinal mechanism is motor overflow or motor irradiation, which refers to unintended extraneous muscle activity. The unintended muscle activity from motor overflow can also lead to involuntary movements, or mirror movements [37]. Motor overflow tends to parallel the level of effort, with high levels of effort or more complex tasks producing greater amounts of unintended muscle activation [38-40]. We also observed a graded effect, where greater levels of effort resulted in greater increases in passive muscle activity during our experimental studies [24,26]. While most motor overflow studies focus on just the upper limbs or just the hands [41], the effect extends beyond just between the arms or hands and can manifest among all four limbs [42,43], similar to our experimental observations [24-27]. Proposed theories to explain motor overflow are supraspinal and suggest coincidental cortical activation and/or activity in the corticospinal projections of unintended muscles [41].

## Conclusions

We used simple computer simulations to model interlimb spinal pathways to test whether spinal neural mechanisms could explain excitatory coupling of muscle recruitment between upper and lower limbs. Interconnecting four CPGs to have symmetrical excitatory ipsilateral flexor-extensor coupling, excitatory contralateral flexor-flexor/extensor-extensor coupling, and inhibitory bilateral flexor-extensor/extensor-flexor coupling produced enhancement up to 32%. This four-CPG model was more sensitive to small changes in interlimb coupling gain compared to the four-CPG model with ipsilateral flexor-extensor/extensor-flexor, contralateral flexor-flexor/extensor-extensor, and bilateral flexor-flexor/extensor-extensor coupling. Enhancement of 32% was not sufficient to explain experimental data that attained levels of enhancement of 50+%. This suggests that symmetrical excitatory interlimb coupling alone could account for much, but not all of the enhancement. By using computer simulations, we could test different neural architectures which were more difficult to investigate experimentally. The computer simulations revealed that a) excitatory ipsilateral coupling acted between flexor-extensor pairs, b) excitatory contralateral coupling acted between flexor-flexor and extensor-extensor pairs, c) bilateral flexor-extensor coupling in a four-CPG was more sensitive to relatively smaller interlimb coupling gains than bilateral flexor-flexor/extensor-extensor coupling, and d) relatively small inhibitory sensory feedback gains entrained CPG rhythmic bursts compared to excitatory sensory feedback gains. These simulations provided insight into the neural mechanisms involved in excitatory interlimb coupling and could help design future experiments to better understand the neural mechanisms of excitatory neural coupling.

## Competing interests

The authors declare that they have no competing interests.

## Authors' contributions

HJH and DPF jointly conceived of the study and its design. HJH built the models, analyzed the simulations, and drafted the manuscript. DPF provided guidance on data analysis and helped edit the manuscript. All authors read and approved the final manuscript.
